# Pyrosequencing for Mini-Barcoding of Fresh and Old Museum Specimens

**DOI:** 10.1371/journal.pone.0021252

**Published:** 2011-07-27

**Authors:** Shadi Shokralla, Xin Zhou, Daniel H. Janzen, Winnie Hallwachs, Jean-François Landry, Luke M. Jacobus, Mehrdad Hajibabaei

**Affiliations:** 1 Biodiversity Institute of Ontario, Department of Integrative Biology, University of Guelph, Guelph, Ontario, Canada; 2 Department of Biology, University of Pennsylvania, Philadelphia, Pennsylvania, United States of America; 3 Research Centre, Agriculture and Agri-Food Canada, Ottawa, Ontario, Canada; 4 Department of Biology, Indiana University, Bloomington, Indiana, United States of America; American Museum of Natural History, United States of America

## Abstract

DNA barcoding is an effective approach for species identification and for discovery of new and/or cryptic species. Sanger sequencing technology is the method of choice for obtaining standard 650 bp cytochrome *c* oxidase subunit I (COI) barcodes. However, DNA degradation/fragmentation makes it difficult to obtain a full-length barcode from old specimens. Mini-barcodes of 130 bp from the standard barcode region have been shown to be effective for accurate identification in many animal groups and may be readily obtained from museum samples. Here we demonstrate the application of an alternative sequencing technology, the four-enzymes single-specimen pyrosequencing, in rapid, cost-effective mini-barcode analysis. We were able to generate sequences of up to 100 bp from mini-barcode fragments of COI in 135 fresh and 50 old Lepidoptera specimens (ranging from 53–97 year-old). The sequences obtained using pyrosequencing were of high quality and we were able to robustly match all the tested pyro-sequenced samples to their respective Sanger-sequenced standard barcode sequences, where available. Simplicity of the protocol and instrumentation coupled with higher speed and lower cost per sequence than Sanger sequencing makes this approach potentially useful in efforts to link standard barcode sequences from unidentified specimens to known museum specimens with only short DNA fragments.

## Introduction

DNA sequences have become a major source of information for understanding biodiversity. In particular, DNA barcoding has been employed as a species identification tool based on the premise that a short standardized sequence of the mitochondrial cytochrome *c* oxidase 1 gene (COI) can distinguish the majority of animal species because, in this locus sequence variation between species generally exceeds that within species [1]. Large-scale DNA barcoding projects have now established the effectiveness of this approach [2]. Consequently, DNA barcode reference libraries are being established for all major groups of eukaryotic organisms. Although freshly collected specimens can provide high-quality DNA sequences and are therefore the optimal material for the construction of DNA barcode reference libraries, museum collections are critical to linking unidentified biodiversity to available taxonomic knowledge- joining our taxonomic legacy with a future in which dedicated full-time taxonomists will be even more rare than today. However, methods used for preservation of museum specimens are often not DNA-friendly [3]. Thus, DNA degradation has been recognized as a considerable limitation for the utility of museum specimens in DNA-based analyses. In fact, the success in obtaining full-length barcodes from old museum specimens (i.e. >10 of age years for dried pinned insects) is almost always significantly lower than that from fresh samples of those same species [4]. Alternatively, a mini-barcoding approach, which focuses the analysis on shorter DNA fragments, has been shown to be effective in gaining DNA sequence information from old museum samples and a 130 bp fragment from the 5′ end of the full-length DNA barcode region has shown to be effective in distinguishing up to 91% of animal species in a broad taxonomic range [4], [5]. This same fragment has even shown promise for DNA analysis in benthic insect collections treated with formalin [6].

The relative ease in obtaining mini-barcodes coupled with the availability of DNA sequencing technologies alternative to Sanger sequencing (i.e., pyrosequencing) [7] provides a new tool to obtain DNA barcode information from samples that often fail to generate full-length barcodes. This approach may also be technically beneficial because sequencing short fragments and GC-rich regions is sometimes challenging for classic Sanger sequencing workflow [8].

Other studies have shown the applicability of real time pyrosequencing for microbial identification including bacterial, fungal, and viral pathogens [9], [10], . In the present study, we assess the potential of applying pyrosequencing technology in the acquisition of mini-barcodes from fresh and old museum Lepidoptera specimens of a wide range of ages. We compare the results obtained by pyrosequencing to Sanger sequencing and discuss pyroseqencing read-length and error rate and their potential influence on species identification.

## Results

Out of 141 DNA extracts of fresh Lepidoptera, 135 (95.7%) and 139 (98.6%) mini-barcode sequences were obtained using pyrosequencing and Sanger sequencing, respectively. Additionally, 50 (90.9%) and 52 (94.5%) out of 55 DNA extracts of older museum Lepidoptera specimens could be mini-barcoded by pyrosequencing and Sanger sequencing, respectively (Table 1).

**Table 1:** Comparisons of DNA Pyrosequencing and Sanger sequencing results of the COI mini-barcode region.

Group	Number of specimens	%PCR success	%Pyrosequencing success	%Sanger sequencing success
Fresh Lepidoptera	141	99.3	95.7	98.6
Old museum Lepidoptera	55	96.4	90.9	94.5

**Figure 1:**
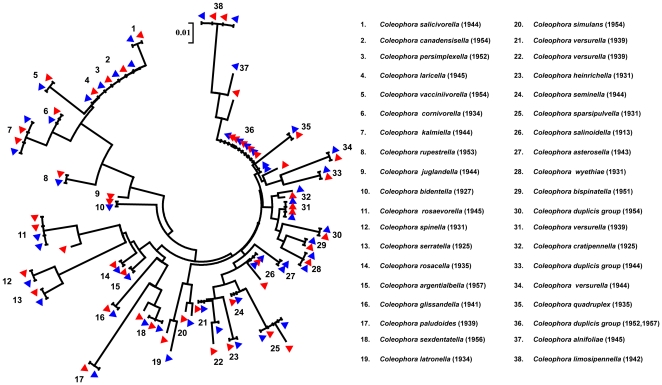
Neighbor-joining (NJ) trees based on Kimura-2-Parameter (K2P) distances for cytochrome *c* oxidase I (COI) mini-barcodes of old museum Lepidoptera specimens. The red markers indicate the position of generated pyrosequences in comparison to the reference library, with blue markers, from BOLD or Genbank. Collection dates are shown in parentheses.

We evaluated the quality of consensus pyrosequences by comparing them to a reference library generated by Sanger sequencing for all Lepidoptera samples. Overall, pyrosequence reads showed high fidelity to reference sequences, where approximately 96.3% and 96.2% of pyrosequences showed >98% similarity to references in fresh and old museum Lepidoptera, respectively. The differences between pyrosequences and the reference Sanger sequences were mostly due to insertions and deletions (indels) introduced by the pyrosequencing chemistry. However, the pyrosequencing reads corresponded to reference sequences of target species through NCBI's Megablast program or by constructing a neighbour-joining tree together with reference sequences (Figure 1). A summary of pyrosequence read lengths generated through *denovo* nucleotide dispensation order and their quality (measured by percentage similarity to reference sequences) is available in the Supplementary Material (Tables S1 and S2).

As for time efficiency, pyrosequencing technique generated 24 pyrograms from 24 biotin labeled PCR products in less than 2 hours using a 24-sample format pyrosequencing platform. The resulting pyrograms can be automatically translated to FASTA format using the embedded PyroMark (SQA) software.

## Discussion

DNA quality is a key limiting aspect of the success of PCR amplification reactions. Long-term preservation of biological samples may cause DNA shearing and DNA inter-strand cross-linking, which consequently result in DNA degradation. DNA shearing is the break-down of DNA into small fragments, which might be introduced by poor storage conditions such as exposure to UV radiation, high temperature, pH, and salinity [3]. Consequently, the probability of obtaining long (i.e. >600 bp) PCR amplicons is much lower for museum specimens or processed biological materials such as food products or natural health products. Short mini-barcodes have been proposed as a cost-effective solution for gaining DNA sequence information in cases where genetic information from samples with degraded DNA is desired [4], [5]. For example, integrative taxonomic studies may benefit greatly from the availability of mini-barcode sequence data in reference panels that include old and historically important specimens, such as those from the original type series. Such data potentially will allow for more confident applications of established names, especially in cases where species names have been considered synonymous because they are based on a cryptic stage of the life cycle. This may reduce the number of *nominadubia* applicable to species whose type specimens are still in existence, if the specimens can be analyzed in a way that minimizes disruption of specimen integrity. The majority of names applicable to eukaryotic life have been established based on specimens younger than our oldest specimen that allowed successful barcode data retrieval. Pyrosequencing, which produces shorter sequence fragments as compared to Sanger sequencing, could be particularly useful for implementing this workflow.

Our comparative analysis of pyrosequencing and Sanger sequencing suggests that the quality of pyrosequencing reads is promising for effective identification at species level. However, we did observe differences between pyrosequences and reference sequences in some cases of our sampling set. These differences are associated to a known issue in pyrosequencing: interpreting homo-polymeric regions is a challenge for pyrosequencing due to over- and under-base calling mainly associated with poly-“A”s and poly-“T”s [8]. The automated base caller in pyrosequencer software is based on the intensity of light signals in the pyrogram, which could mislead base calling when a homopolymer is encountered. This will result in ambiguity of homopolymer length, especially for relatively longer homopolymers. On the other hand, insufficient nucleotide incorporation within a flow can cause incomplete extension within homopolymers which can lead to under-base calling [12]. Such artifacts, however, can be detected and corrected in protein-coding genes, such as COI barcode, by examining the amino acid translation frame and by comparing pyrosequences to arrays of reference sequences. Additionally, bioinformatics solutions for homopolymer detection can further reduce the negative impact of this issue on data quality. Thus, the indels observed in this study should not represent a major issue for the implementation of pyrosequencing in mini-barcode applications.

The pyrosequencing approach showed comparable sequencing success to the gold standard Sanger sequencing for both fresh and museum specimens. As PCR amplification of the targets is a critical step prior to any sequencing approach, caution should be taken in amplification of non-target amplicons especially from old museum DNA samples. Thus, the use of specific primer sets designed and optimized for each taxonomic group may increase both PCR and sequencing success rates. However, the formation of chimeric sequences during PCR step due to aborted extension products which can interfere with the traditional Sanger sequencing [13], is not a problem in the pyrosequencing workflow as one of the amplification primers is biotin-labeled, thus only complete biotin labeled amplicons will be successfully sequenced and all chimeric sequences will be either washed off or will fail to anneal to the pyrosequencing primer. Moreover, another advantage of pyrosequencing is the ability to sequence more than one variable region from the same amplicon using various sequencing primers that are specifically designed for certain regions. Thus, pyrosequencing provides a rapid and inexpensive method to distinguish closely related species where only few nucleotides at certain loci are different [14].

**Figure 2:**
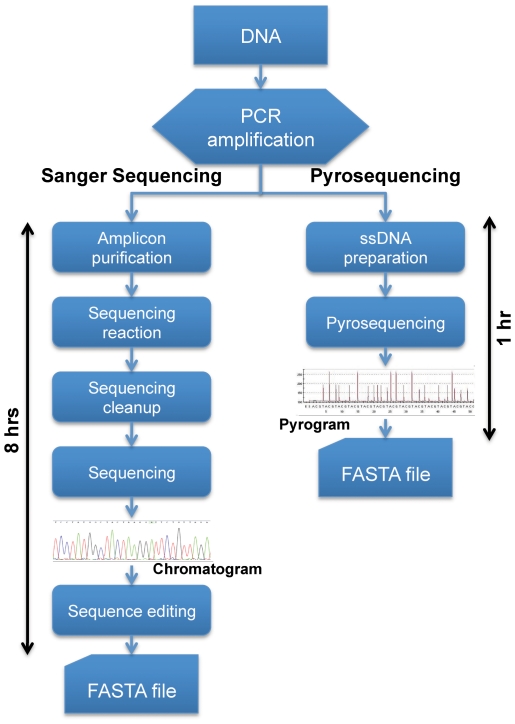
Schematic representation of pyrosequencing vs. Sanger sequencing workflows.

In addition to its advantages in the PCR amplification and sequencing protocols, the pyrosequencing pipeline is more time-efficient. As pyrosequencing requires fewer stages and the sequence detection is done in real-time, therefore, it is a faster approach as compared to classic Sanger sequencing (approximately 2 hrs. for pyrosequencing vs. 8–10 hrs. for Sanger sequencing; Figure 2). Furthermore, the pyrosequencing laboratory requires only a basic thermocycler and a pyrosequencing station. Additionally, only minimal expertise is required in pyrosequencing laboratory protocols and the overall pipeline is less labor-intensive and more cost-effective (less than 1$ per sequence) as compared to Sanger sequencing.

In conclusion, although Sanger sequencing remains the workhorse for building conventional full-length DNA barcode libraries, pyrosequencing technology provides a simple, rapid, and inexpensive alternative method to generate mini-barcodes in both fresh and museum samples and other biological material with potentially degraded DNA. Because of these properties, pyrosequencing is suitable in situations where a full-fledged molecular laboratory is not a feasible option. For example, this approach can be readily implemented in small laboratories built in a museum setting.

## Materials and Methods

### Specimens and taxonomic coverage

DNA extracts of Lepidoptera (moths and butterflies) specimens were obtained from many DNA barcoding projects conducted at the Biodiversity Institute of Ontario, University of Guelph, using routine DNA extraction protocols [15]. Specimens were selected to maximize taxonomic representation and age range. Fresh Lepidoptera specimens were obtained from Area de Conservación Guanacaste (ACG) in northwestern Costa Rica as part of a large-scale bio-inventory project that has been using DNA barcoding for species identification and discovery [16], [17]. These specimens were mainly collected in the last 10 years. The old Lepidoptera samples (ranging from 53 to 97 years old) were obtained from the Canadian National Collection of Insects, Arachnids, and Nematodes (CNC). Detailed sample information is available in the Supplementary Material (Tables S1 and S2).

### Primer selection and modification strategy

A total of 141 DNA extracts from recently collected (≤4 years old) Lepidoptera and 55 DNA extracts from old (≥53 years) Lepidoptera specimens were PCR amplified for the COI mini-barcode region [5] and were subsequently subjected to both pyro- and Sanger sequencing in parallel. Primers used in routine barcoding protocols, i.e., LepF [16], [17], LCO1490 [18], and minibar-UnivR [4], were modified in two ways. First, either the forward or reverse amplification primer in each primer set is biotin-labeled on the 5′ end. Second, 3 “N”s were added to the 5′ end or one or two nucleotides were trimmed from the 3′ end of the sequencing primers to achieve the best annealing to the single stranded template to fit the pyrosequencing protocol (Table 2). All primers were tested using IDT oligo analyzer web tool (http://www.idtdna.com/analyzer/Applications/OligoAnalyzer/) considering their physical and structural properties.

**Table 2:** Primers used for PCR amplification and pyrosequencing.

Primer code	Sequence (5′- 3′)
Amplification primers	
LCO1490F	GGTCAACAAATCATAAAGATATTGG
LepF	ATTCAACCAATCATAAAGATATTGG
Minibar-UnivR	GAAAATCATAATGAAGGCATGAGC
Sequencing primers	
Pyro_LCO1490F	NNNGTCAACAAATCATAAAGATATTG
Pyro_LepF	NNNATTCAACCAATCATAAAGATATTGG
Pyro_Minibar-UnivR	NNNGAAAATCATAATGAAGGCATGA

### PCR optimization strategy

PCRs were assembled in 25 µl reactions each containing 2 µl DNA template, 17.5 µl molecular biology grade water, 2.5 µl 10× invitrogen buffer, 1 µl 50× MgCl2 (50 mM), 0.5 µl dNTPs mix (10 mM), 0.5 µl forward primer (10 mM), 0.5 µl reverse primer (10 mM), and 0.5 µl Invitrogen Platinum Taq polymerase (5 U/µl). The touchdown PCR conditions were initiated with heated lid at 95°C for 5 min, followed by 15 cycles of 94°C for 40 sec, 55°C for 1 min, and 72°C for 30 sec, followed by 30 cycles of 94°C for 40 sec, 46°C for 1 min, and 72°C for 30 sec, a final extension at 72°C for 5 min, and hold at 4°C. We used a Mastercyclerep gradient S (Eppendorf, Mississauga, ON, Canada) thermal cycler. A negative control reaction (no DNA template) was included in all experiments.

### Pyrosequencing

Pyrosequencing was performed in the Qiagen Pyromark ID platform following manufacturer's instructions using PyroMark Gold Q96 SQA Reagents with some modifications as follows. All generated amplicons including the negative controls were immobilized to streptavidin coated sepharose beads by shaking at 1400 rpm for 10 min. Double-strand DNA (amplicons) was then denatured to single-stranded DNA (ssDNA) using a denaturing (0.5 N NaOH) buffer and Qiagen vacuum preparation workstation. Single-stranded DNA was then annealed to a specific sequencing primer (Table 2) at 80°C for 2 min. For each sequencing reaction, 2 µl of enzyme mixture and 2 µl of substrate mixture were added directly to the wells of the pyrosequencing plate instead of the dispensing cassette. The sequencing reactions were loaded on the PyroMark ID system equipped with PyroMark ID software for pyrosequencing set with 100 *de-novo* nucleotide dispenses. The sample was considered successful in Pyrosequencing when high quality sequences could be generated from both forward and reverse directions indicating specific target amplification in the PCR step.

### Pyrosequencing data analysis

The generated pyrograms were automatically analyzed using the PyroMark analysis software. All pyrograms were revised and signal intensities were processed automatically by PyroMark sequence analysis (SQA) software so that the bases were assigned either as “good quality” or “check quality”. The generated sequences were exported in FASTA format. Both forward and reverse sequences were used to assemble contigs using MEGA V.4.0 [19]. Sequence quality of the pyrosequencing reads was examined by comparing consensus pyrosequences to reference DNA barcodes obtained from the same sets of specimens previously generated using Sanger sequencing. When Sanger mini-barcode sequences were not available from the same specimens, the obtained pyrosequences were compared to barcode libraries available in the Barcode of Life Data System (BOLD) [20] or GenBank. All Sanger and pyrosequencing mini-barcodes as well as details about the tested samples are available by request from the corresponding author.

## Supporting Information

Table S1 Fresh Lepidoptera specimens used for testing the Pyrosequencing approach for COI mini-barcodes.
(DOCX)

Table S2 Old museum Lepidoptera specimens used for testing the Pyrosequencing approach for COI mini-barcodes.
(DOCX)

## References

[1] Hebert PDN, Cywinska A, Ball SL, deWaard JR (2003). Biological identifications through DNA barcodes.. Proceedings of the Royal Society of London B Biological Sciences.

[2] Hajibabaei M, Singer GAC, Hebert PDN, Hickey DA (2007). DNA barcoding: how it complements taxonomy, molecular phylogenetics and population genetics.. Trends Genet.

[3] Mandrioli M, Borsatti F, Mola L (2006). Factors affecting DNA preservation from museum-collected lepidopteran specimens.. Entomologia Experimentalis et Applicata.

[4] Hajibabaei M, Smith MA, Janzen DH, Rodriguez JJ, Whitfield JB (2006). A minimalist barcode can identify a specimen whose DNA is degraded.. Molecular Ecology Notes.

[5] Meusnier I, Singer GAC, Landry JF, Hickey DA, Hebert PDN (2008). A universal DNA mini-barcode for biodiversity analysis.. BMC Genomics.

[6] Baird DJ, Pascoe TJ, Zhou X, Hajibabaei M (2011). Building freshwater macroinvertebrate DNA barcode libraries from reference collection material: formalin preservation versus specimen age.. Journal of the North American Benthological Society.

[7] Ronaghi M, Uhlen M, Nyren P (1998). A sequencing method based on real-time pyrophosphate.. Science.

[8] Gharizadeh B, Herman Z, Eason R, Jejelowo O, Pourmand N (2006). Large-scale Pyrosequencing of synthetic DNA: A comparison with results from Sanger dideoxy sequencing.. Electrophoresis.

[9] Adelson ME, Feola M, Trama J, Tilton RC, Mordechai E (2005). Simultaneous detection of herpes simplex virus types 1 and 2 by real-time PCR and Pyrosequencing.. J Clin Virol.

[10] Gharizadeh B, Norberg E, Löffler J, Jalal S, Tollemar J (2004). Identification of medically important fungi by the Pyrosequencing technology.. Mycoses.

[11] Jordan JA, Butchko AR, Durso MB (2005). Use of pyrosequencing of 16S rRNA fragments to differentiate between bacteria responsible for neonatal sepsis.. J Mol Diagn.

[12] Huse SM, Huber JA, Morrison HG, Sogin ML, Welch DM (2007). Accuracy and quality of massively parallel DNA pyrosequencing.. Genome Biol.

[13] Haas B, Gevers D, Earl A, Feldgarden M, Ward D (2011). Chimeric 16S rRNA sequence formation and detection in Sangerand 454-pyrosequenced PCR amplicons.. Genome Res.

[14] Ronaghi M, Shokralla S, Gharizadeh B (2007). Pyrosequencing for discovery and analysis of DNA sequence variations.. Pharmacogenomics.

[15] Ivanova N, deWaard J, Hebert PDN (2006). An inexpensive, automation-friendly protocol for recovering high-quality DNA.. Molecular Ecology Notes.

[16] Hajibabaei M, Janzen DH, Burns JM, Hallwachs W, Hebert PDN (2006). DNA barcodes distinguish species of tropical Lepidoptera.. Proceedings of the National Academy of Sciences of the United States of America.

[17] Janzen DH, Hajibabaei M, Burns JM, Hallwachs W, Remigio E (2005). Wedding biodiversity inventory of a large and complex Lepidoptera fauna with DNA barcoding.. Philosophical Transactions of the Royal Society of London B Biological Sciences.

[18] Folmer O, Black M, Hoeh W, Lutz R, Vrijenhoek R (1994). DNA primers for amplification of mitochondrial cytochrome C oxidase subunit I from diverse metazoan invertebrates.. Molecular Marine Biology and Biotechnology.

[19] Tamura K, Dudley J, Nei M, Kumar S (2007). MEGA4: Molecular Evolutionary Genetics Analysis (MEGA) software version 4.0.. Mol Biol Evol.

[20] Ratnasingham S, Hebert PDN (2007). BOLD: The Barcode of Life Data System (www.barcodinglife.org).. Molecular Ecology Notes.

