# Supplementary material for: Pyrosequencing for Mini-Barcoding of Fresh and Old Museum Specimens
Source: PLoS One. 2011 Jul 27;6(7):e21252. doi: 10.1371/journal.pone.0021252 (PMC3144868; doi:10.1371/journal.pone.0021252)
Supplement: Table S1 — Fresh Lepidoptera specimens used for testing the Pyrosequencing approach for COI mini-barcodes. (DOCX) [file pone.0021252.s001.docx]

**Supplementary material**

Table S1. Fresh Lepidoptera specimens used for testing the Pyrosequencing approach for COI mini-barcodes

| **Specimen#** | **Samples**  **ID** | **Taxonomy** | | **Age**  **(year)** | **Forward (bp)** | **Reverse (bp)** | **% of identity** | **Un-sequenced nucleotides** |
| --- | --- | --- | --- | --- | --- | --- | --- | --- |
|  |  | **Family** | **Genus, Species** |  |  |  |  |  |
| **1** | 08-SRNP-41661 | Lasiocampidae | Euglyphis lepta | 1.86 | 65 | 65 | 98.5 | 9 |
| **2** | 08-SRNP-5075 | Lasiocampidae | Euglyphis Montero04 | 1.82 | 72 | 83 | 100 | 0 |
| **3** | 08-SRNP-5074 | Lasiocampidae | Euglyphis asapha | 1.88 | 66 | 64 | 100 | 5 |
| **4** | 08-SRNP-30940 | Lasiocampidae | Euglyphis lepta | 2.35 | 59 | 72 | 100 | 0 |
| **5** | 07-SRNP-14494 | Lasiocampidae | Euglyphis phyllisDHJ02 | 3.01 | 60 | 72 | 100 | 0 |
| **6** | 08-SRNP-2248 | Sematuridae | Coronidia subpicta | 2.23 | 66 | 46 | 100 | 18 |
| **7** | 08-SRNP-36535 | Sematuridae | Coronidia subpicta | 2.23 | 62 | 49 | 100 | 21 |
| **8** | 08-SRNP-2250 | Sematuridae | Coronidia subpicta | 2.23 | 63 | 65 | 99.2 | 14 |
| **9** | 08-SRNP-2249 | Sematuridae | Coronidia subpicta | 2.23 | No Pyrosequence | | | |
| **10** | 08-SRNP-2240 | Sematuridae | Coronidia subpicta | 2.23 | 63 | 76 | 100 | 0 |
| **11** | 08-SRNP-36940 | Sematuridae | Coronidia subpicta | 1.78 | 45 | 85 | 95.4 | 0 |
| **12** | 08-SRNP-37044 | Sematuridae | Coronidia subpicta | 1.78 | 64 | 66 | 100 | 14 |
| **13** | 08-SRNP-36533 | Sematuridae | Coronidia subpicta | 1.84 | 60 | 83 | 100 | 0 |
| **14** | 08-SRNP-30952 | Lasiocampidae | Euglyphis asapha | 1.1 | 66 | 64 | 100 | 5 |
| **15** | 08-SRNP-30953 | Lasiocampidae | Euglyphis asapha | 1.1 | 61 | 86 | 100 | 0 |
| **16** | 08-SRNP-14248 | Lasiocampidae | Tytocha lineate | 2 | 81 | 55 | 100 | 0 |
| **17** | 08-SRNP-71150 | Lasiocampidae | Euglyphis lepta | 1.1 | 65 | 72 | 100 | 0 |
| **18** | 08-SRNP-30761 | Lasiocampidae | Euglyphis lepta | 2.34 | 67 | 67 | 100 | 0 |
| **19** | 08-SRNP-30946 | Lasiocampidae | Euglyphis lepta | 2.35 | 63 | 67 | 99.2 | 6 |
| **20** | 08-SRNP-30944 | Lasiocampidae | Euglyphis lepta | 2.35 | 53 | 76 | 96.9 | 5 |
| **21** | 08-SRNP-41660 | Lasiocampidae | Euglyphis lepta | 1.87 | No Pyrosequence | | | |
| **22** | 08-SRNP-41662 | Lasiocampidae | Euglyphis lepta | 1.85 | No Pyrosequence | | | |
| **23** | 08-SRNP-4470 | Lasiocampidae | Euglyphis lepta | 1.96 | 70 | 60 | 100 | 7 |
| **24** | 08-SRNP-35517 | Lasiocampidae | Euglyphis Janzen01 | 2.12 | 81 | 75 | 100 | 0 |
| **25** | 08-SRNP-45160 | Lasiocampidae | Euglyphis Janzen01 | 2.25 | 66 | 84 | 100 | 0 |
| **26** | 08-SRNP-57379 | Lasiocampidae | Euglyphis Janzen01 | 1.93 | 88 | 42 | 100 | 10 |
| **27** | 08-SRNP-6277 | Lasiocampidae | Euglyphis Janzen01 | 1.61 | 69 | 66 | 100 | 0 |
| **28** | 08-SRNP-56538 | Lasiocampidae | Euglyphis Janzen01 | 2.04 | 65 | 80 | 100 | 0 |
| **29** | 08-SRNP-610 | Lasiocampidae | Euglyphis Janzen01 | 2.41 | 67 | 68 | 100 | 0 |
| **30** | 08-SRNP-1565 | Lasiocampidae | Euglyphis Janzen01 | 2.31 | 53 | 87 | 100 | 0 |
| **31** | 08-SRNP-57448 | Lasiocampidae | Euglyphis Janzen01 | 1.89 | 70 | 83 | 99.2 | 0 |
| **32** | 08-SRNP-57454 | Lasiocampidae | Euglyphis Janzen01 | 1.91 | 64 | 66 | 100 | 5 |
| **33** | 08-SRNP-57710 | Lasiocampidae | Euglyphis Janzen01 | 1.82 | 73 | 66 | 100 | 0 |
| **34** | 08-SRNP-5949 | Lasiocampidae | Euglyphis Janzen01 | 1.69 | 63 | 68 | 100 | 5 |
| **35** | 08-SRNP-57628 | Lasiocampidae | Euglyphis Janzen01 | 1.81 | No Pyrosequence | | | |
| **36** | 08-SRNP-4712 | Lasiocampidae | Euglyphis Janzen01 | 1.9 | 59 | 73 | 100 | 0 |
| **37** | 08-SRNP-57386 | Lasiocampidae | Euglyphis Janzen01 | 1.91 | 64 | 66 | 100 | 7 |
| **38** | 08-SRNP-609 | Lasiocampidae | Euglyphis Janzen01 | 2.41 | 63 | 85 | 100 | 0 |
| **39** | 08-SRNP-2099 | Lasiocampidae | Euglyphis Janzen01 | 2.25 | 65 | 71 | 98.5 | 0 |
| **40** | 08-SRNP-1724 | Lasiocampidae | Euglyphis Montero04 | 2.28 | 56 | 86 | 99.2 | 0 |
| **41** | 08-SRNP-1721 | Lasiocampidae | Euglyphis Montero04 | 2.29 | 93 | 42 | 100 | 0 |
| **42** | 08-SRNP-1726 | Lasiocampidae | Euglyphis Montero04 | 2.26 | 49 | 83 | 99.2 | 0 |
| **43** | 08-SRNP-4193 | Lasiocampidae | Euglyphis fibra | 2.04 | 65 | 73 | 100 | 0 |
| **44** | 08-SRNP-4190 | Lasiocampidae | Nesara casada | 2.05 | 56 | 75 | 100 | 0 |
| **45** | 08-SRNP-5690 | Lasiocampidae | Nesara casada | 1.77 | 75 | 82 | 99.2 | 0 |
| **46** | 08-SRNP-5148 | Lasiocampidae | Nesara casada | 1.86 | 63 | 68 | 100 | 6 |
| **47** | 08-SRNP-5464 | Lasiocampidae | Nesara casada | 1.81 | 71 | 61 | 97.7 | 9 |
| **48** | 08-SRNP-57480 | Lasiocampidae | Euglyphis phyllisDHJ02 | 1.89 | 76 | 66 | 100 | 0 |
| **49** | 07-SRNP-14499 | Lasiocampidae | Euglyphis phyllisDHJ02 | 3.01 | 63 | 49 | 100 | 19 |
| **50** | 07-SRNP-14500 | Lasiocampidae | Euglyphis phyllisDHJ02 | 3.01 | 71 | 60 | 100 | 12 |
| **51** | 07-SRNP-16165 | Lasiocampidae | Euglyphis phyllisDHJ01 | 2.74 | 88 | 43 | 99.2 | 0 |
| **52** | 07-SRNP-16491 | Lasiocampidae | Euglyphis phyllisDHJ01 | 2.63 | 76 | 71 | 100 | 0 |
| **53** | 07-SRNP-16492 | Lasiocampidae | Euglyphis phyllisDHJ01 | 2.62 | 66 | 72 | 100 | 0 |
| **54** | 07-SRNP-16490 | Lasiocampidae | Euglyphis phyllisDHJ01 | 2.63 | 82 | 65 | 100 | 0 |
| **55** | 07-SRNP-15219 | Lasiocampidae | Euglyphis phyllisDHJ01 | 2.95 | 70 | 62 | 100 | 5 |
| **56** | 07-SRNP-14425 | Lasiocampidae | Euglyphis phyllisDHJ01 | 3.03 | 67 | 63 | 99.2 | 6 |
| **57** | 07-SRNP-14449 | Lasiocampidae | Euglyphis phyllisDHJ01 | 3.02 | 77 | 62 | 100 | 0 |
| **58** | 07-SRNP-14575 | Lasiocampidae | Euglyphis phyllisDHJ01 | 3.01 | 83 | 47 | 100 | 5 |
| **59** | 07-SRNP-16494 | Lasiocampidae | Euglyphis phyllisDHJ01 | 2.65 | 51 | 92 | 100 | 0 |
| **60** | 08-SRNP-35149 | Lasiocampidae | Euglyphis lankesteri | 2.29 | 65 | 71 | 100 | 0 |
| **61** | 08-SRNP-35153 | Lasiocampidae | Euglyphis lankesteri | 2.29 | 86 | 82 | 100 | 0 |
| **62** | 08-SRNP-35152 | Lasiocampidae | Euglyphis lankesteri | 1.96 | 63 | 71 | 100 | 0 |
| **63** | 08-SRNP-35151 | Lasiocampidae | Euglyphis lankesteri | 2.29 | 80 | 69 | 100 | 0 |
| **64** | 08-SRNP-35154 | Lasiocampidae | Euglyphis lankesteri | 2.29 | 63 | 77 | 100 | 0 |
| **65** | 08-SRNP-56264 | Lasiocampidae | Euglyphis gutturalis | 2.11 | 76 | 54 | 100 | 7 |
| **66** | 08-SRNP-57477 | Lasiocampidae | Euglyphis gutturalis | 1.91 | 89 | 41 | 99.2 | 6 |
| **67** | 08-SRNP-55402 | Lasiocampidae | Euglyphis gutturalis | 2.49 | 73 | 64 | 100 | 0 |
| **68** | 08-SRNP-4433 | Lasiocampidae | Artace cribraria | 2 | No Pyrosequence | | | |
| **69** | 08-SRNP-36857 | Lasiocampidae | Euglyphis deustaDHJ02 | 1.72 | 77 | 60 | 99.2 | 0 |
| **70** | 08-SRNP-36817 | Lasiocampidae | Euglyphis deustaDHJ02 | 1.77 | 73 | 64 | 100 | 0 |
| **71** | 08-SRNP-36922 | Lasiocampidae | Euglyphis deustaDHJ02 | 1.7 | 69 | 65 | 100 | 0 |
| **72** | 08-SRNP-36927 | Lasiocampidae | Euglyphis deustaDHJ02 | 1.7 | 66 | 75 | 100 | 0 |
| **73** | 08-SRNP-35378 | Lasiocampidae | Euglyphis deustaDHJ02 | 2.19 | 89 | 54 | 100 | 0 |
| **74** | 08-SRNP-35375 | Lasiocampidae | Euglyphis deustaDHJ02 | 2.21 | 71 | 64 | 100 | 0 |
| **75** | 08-SRNP-36818 | Lasiocampidae | Euglyphis deustaDHJ02 | 1.77 | 87 | 51 | 100 | 0 |
| **76** | 08-SRNP-35377 | Lasiocampidae | Euglyphis deustaDHJ02 | 2.19 | 69 | 73 | 100 | 0 |
| **77** | 08-SRNP-35472 | Lasiocampidae | Euglyphis deustaDHJ02 | 2.19 | 66 | 71 | 100 | 0 |
| **78** | 08-SRNP-35379 | Lasiocampidae | Euglyphis deustaDHJ02 | 2.21 | 58 | 72 | 98.5 | 0 |
| **79** | 08-SRNP-36870 | Lasiocampidae | Euglyphis deustaDHJ02 | 1.73 | 78 | 65 | 100 | 0 |
| **80** | 08-SRNP-35401 | Lasiocampidae | Euglyphis deustaDHJ02 | 2.2 | 91 | 54 | 100 | 0 |
| **81** | 08-SRNP-35407 | Lasiocampidae | Euglyphis deustaDHJ02 | 2.2 | 45 | 77 | 99.2 | 8 |
| **82** | 08-SRNP-35461 | Lasiocampidae | Euglyphis deustaDHJ02 | 2.16 | 62 | 68 | 100 | 6 |
| **83** | 08-SRNP-35406 | Lasiocampidae | Euglyphis deustaDHJ02 | 2.21 | 69 | 67 | 100 | 0 |
| **84** | 08-SRNP-35460 | Lasiocampidae | Euglyphis deustaDHJ02 | 2.18 | 82 | 48 | 96.9 | 0 |
| **85** | 08-SRNP-35411 | Lasiocampidae | Euglyphis deustaDHJ02 | 2.2 | 71 | 66 | 100 | 0 |
| **86** | 08-SRNP-35400 | Lasiocampidae | Euglyphis deustaDHJ02 | 2.19 | 75 | 63 | 100 | 0 |
| **87** | 08-SRNP-35398 | Lasiocampidae | Euglyphis deustaDHJ02 | 2.21 | 77 | 65 | 99.2 | 0 |
| **88** | 08-SRNP-35399 | Lasiocampidae | Euglyphis deustaDHJ02 | 2.2 | 63 | 88 | 100 | 0 |
| **89** | 08-SRNP-35462 | Lasiocampidae | Euglyphis deustaDHJ02 | 2.19 | 62 | 83 | 99.2 | 0 |
| **90** | 08-SRNP-35397 | Lasiocampidae | Euglyphis deustaDHJ02 | 2.18 | 65 | 72 | 100 | 0 |
| **91** | 08-SRNP-1699 | Lasiocampidae | Euglyphis deustaDHJ02 | 2.25 | 63 | 73 | 100 | 0 |
| **92** | 08-SRNP-35412 | Lasiocampidae | Euglyphis deustaDHJ02 | 2.2 | 69 | 68 | 100 | 0 |
| **93** | 08-SRNP-35402 | Lasiocampidae | Euglyphis deusta | 2.2 | 71 | 69 | 100 | 0 |
| **94** | 08-SRNP-1700 | Lasiocampidae | Euglyphis deusta | 2.23 | 82 | 53 | 100 | 0 |
| **95** | \| 08-SRNP-55675 \| \| --- \| | Hesperiidae | Phocides belus | 2.1 | 63 | 67 | 99.2 | 6 |
| **96** | 08-SRNP-70790 | Hesperiidae | Saliana esperi | 2.2 | 72 | 64 | 100 | 0 |
| **97** | 08-SRNP-2301 | Hesperiidae | Talides sergestus | 2.1 | 73 | 91 | 100 | 0 |
| **98** | 08-SRNP-56001 | Hesperiidae | Talides sinois | 2.6 | 63 | 81 | 100 | 0 |
| **99** | 08-SRNP-70314 | Hesperiidae | Elbella Scylla | 2.1 | 68 | 70 | 100 | 0 |
| **100** | 08-SRNP-70328 | Hesperiidae | Pyrrhopyge zenodorus | 2.1 | 72 | 88 | 100 | 0 |
| **101** | 08-SRNP-1660 | Hesperiidae | Phocides lilea | 2.2 | 81 | 93 | 100 | 0 |
| **102** | 08-SRNP-55431 | Hesperiidae | Mysoria ambigua | 2.2 | 58 | 83 | 100 | 0 |
| **103** | 08-SRNP-20448 | Hesperiidae | Salatis canalis | \|  2.2 \| \| --- \| | 65 | 70 | 100 | 0 |
| **104** | 08-SRNP-20672 | Hesperiidae | Bungalotis erythus | 2.2 | 71 | 88 | 100 | 0 |
| **105** | 08-SRNP-70839 | Hesperiidae | Dyscophellus nicephorus | 2.1 | 63 | 67 | 99.2 | 9 |
| **106** | 08-SRNP-21611 | Hesperiidae | Dyscophellus phraxanor | 2.3 | 66 | 72 | 100 | 0 |
| **107** | 08-SRNP-2317 | Hesperiidae | Astraptes fulgerator | 2.2 | 55 | 98 | 100 | 0 |
| **108** | 08-SRNP-4065 | Hesperiidae | Astraptes CELT | 2.2 | 66 | 83 | 100 | 0 |
| **109** | 08-SRNP-1791 | Hesperiidae | Telemiades fides | 2.2 | 53 | 68 | 100 | 9 |
| **110** | 08-SRNP-593 | Hesperiidae | Telemiades antiope | 2 | 69 | 77 | 100 | 0 |
| **111** | 08-SRNP-40908 | Hesperiidae | Vettius pica | 2 | 88 | 0 | 100 | 41 |
| **112** | 08-SRNP-40861 | Hesperiidae | Cephise nuspesez | 2 | 71 | 90 | 100 | 0 |
| **113** | 08-SRNP-1112 | Hesperiidae | Phanus obscurior | 2 | 59 | 99 | 100 | 0 |
| **114** | 08-SRNP-713 | Hesperiidae | Phanus vitreus | 2 | 79 | 88 | 100 | 0 |
| **115** | 07-SRNP-36883 | Hesperiidae | Astraptes creteus crana | 2.9 | 78 | 63 | 99.2 | 0 |
| **116** | 08-SRNP-956 | Hesperiidae | Turesis complanula | 2.6 | 81 | 79 | 100 | 0 |
| **117** | 07-SRNP-13008 | Hesperiidae | Carystoides basoches | 3 | 62 | 96 | 100 | 0 |
| **118** | 08-SRNP-519 | Hesperiidae | Carystoides orbius | 2.2 | 47 | 67 | 100 | 17 |
| **119** | 08-SRNP-45032 | Hesperiidae | Carystoides escalantei | 2.1 | 88 | 83 | 100 | 0 |
| **120** | 08-SRNP-56040 | Hesperiidae | Udranomia kikkawai | 2 | 73 | 57 | 99.2 | 6 |
| **121** | 08-SRNP-71686 | Hesperiidae | Udranomia orcinus | 2 | 63 | 70 | 100 | 0 |
| **122** | 08-SRNP-844 | Hesperiidae | Sostrata bifasciata nordica | 2.1 | 86 | 93 | 100 | 0 |
| **123** | 08-SRNP-794 | Hesperiidae | Quadrus contubernalis | 2.1 | 78 | 92 | 100 | 0 |
| **124** | 08-SRNP-45059 | Hesperiidae | Quadrus cerialis | 2.2 | 69 | 86 | 100 | 0 |
| **125** | 08-SRNP-30973 | Hesperiidae | Thessia jalapus | 2 | 50 | 64 | 98.5 | 16 |
| **126** | 08-SRNP-55989 | Hesperiidae | Urbanus proteus | 1.9 | 89 | 35 | 100 | 8 |
| **127** | 08-SRNP-2053 | Hesperiidae | Polythrix octomaculata | 2.1 | 69 | 68 | 98.5 | 0 |
| **128** | 08-SRNP-2029 | Hesperiidae | Polythrix asine | 2.1 | 85 | 93 | 100 | 0 |
| **129** | 07-SRNP-61073 | Hesperiidae | Aguna panama | 2.9 | 72 | 93 | 100 | 0 |
| **130** | 08-SRNP-31055 | Hesperiidae | Polythrix caunus | 2 | 69 | 60 | 99.2 | 1 |
| **131** | 08-SRNP-70876 | Hesperiidae | Autochton zarex | 2.2 | No Pyrosequence | | | |
| **132** | 07-SRNP-14553 | Hesperiidae | Perichares adela | 2.8 | 71 | 65 | 100 | 0 |
| **133** | 07-SRNP-14581 | Hesperiidae | Perichares philetes complex | 2.8 | 63 | 79 | 100 | 0 |
| **134** | 08-SRNP-71826 | Hesperiidae | Pythonides amaryllis | 2.1 | 76 | 83 | 100 | 0 |
| **135** | 08-SRNP-56931 | Hesperiidae | Zera hosta | 2.1 | 52 | 74 | 96.9 | 6 |
| **136** | 08-SRNP-55841 | Hesperiidae | Clito aberrans | 2.1 | 63 | 67 | 100 | 5 |
| **137** | 08-SRNP-40484 | Hesperiidae | Anastrus neaeris | 2.2 | 79 | 85 | 100 | 0 |
| **138** | 08-SRNP-55465 | Hesperiidae | Aethilla lavochrea | 2.2 | 83 | 69 | 100 | 0 |
| **139** | 08-SRNP-70289 | Hesperiidae | Ebrietas osyris | 2.2 | 91 | 76 | 100 | 0 |
| **140** | 08-SRNP-55673 | Hesperiidae | Chrysoplectrum pervivax | 2.1 | 89 | 73 | 100 | 0 |
| **141** | 08-SRNP-2594 | Hesperiidae | Venada daneva | 2.1 | 75 | 84 | 100 | 0 |
