# Supplementary material for: Pyrosequencing for Mini-Barcoding of Fresh and Old Museum Specimens
Source: PLoS One. 2011 Jul 27;6(7):e21252. doi: 10.1371/journal.pone.0021252 (PMC3144868; doi:10.1371/journal.pone.0021252)
Supplement: Table S2 — Old museum Lepidoptera specimens used for testing the Pyrosequencing approach for COI mini-barcodes. (DOCX) [file pone.0021252.s002.docx]

**Supplementary material**

Table S2. Old museum Lepidoptera specimens used for testing the Pyrosequencing approach for COI mini-barcodes

| **Specimen#** | **Samples ID** | **Taxonomy** | | **Age**  **(year)** | **Forward (bp)** | **Reverse (bp)** | **% of identity** | | **Un-sequenced nucleotides** |
| --- | --- | --- | --- | --- | --- | --- | --- | --- | --- |
|  |  | **Family** | **Genus, Species** |  |  |  |  |  |  |
| **1** | CNCLEP00031133 | Coleophoridae | Coleophora duplicis group | 53 | 94 | 35 | 99.2 | | 0 |
| **2** | CNCLEP00019856 | Coleophoridae | Coleophora duplicis group | 53 | 52 | 78 | 98.5 | | 0 |
| **3** | CNCLEP00031134 | Coleophoridae | Coleophora alnifoliae | 65 | No Pyrosequence | | | | |
| **4** | CNCLEP00031135 | Coleophoridae | Coleophora asterosella | 67 | 52 | 81 | | 98.5 | 0 |
| **5** | CNCLEP00019859 | Coleophoridae | Coleophora asterosella | 67 | No Pyrosequence | | | | |
| **6** | CNCLEP00031138 | Coleophoridae | Coleophora bidentella | 83 | 63 | 88 | | 100 | 0 |
| **7** | CNCLEP00019863 | Coleophoridae | Coleophora bispinatella | 59 | 77 | 100 | | 100 | 0 |
| **8** | CNCLEP00031139 | Coleophoridae | Coleophora canadensisella | 56 | 73 | 64 | | 100 | 0 |
| **9** | CNCLEP00019864 | Coleophoridae | Coleophora canadensisella | 56 | No Pyrosequence | | | | |
| **10** | CNCLEP00019865 | Coleophoridae | Coleophora versurella | 66 | 80 | 75 | | 100 | 0 |
| **11** | CNCLEP00031141 | Coleophoridae | Coleophora kalmiella | 66 | 67 | 51 | | 100 | 9 |
| **12** | CNCLEP00031094 | Coleophoridae | Coleophora cratipennella | 85 | 63 | 69 | | 99.2 | 0 |
| **13** | CNCLEP00031142 | Coleophoridae | Coleophora argentialbella | 53 | 71 | 68 | | 100 | 0 |
| **14** | CNCLEP00019887 | Coleophoridae | Coleophora kalmiella | 58 | 53 | 74 | | 100 | 0 |
| **15** | CNCLEP00031143 | Coleophoridae | Coleophora glissandella | 69 | 57 | 74 | | 100 | 0 |
| **16** | CNCLEP00031144 | Coleophoridae | Coleophora heinrichella | 79 | 63 | 82 | | 100 | 0 |
| **17** | CNCLEP00031145 | Coleophoridae | Coleophora intermediella | 76 | 76 | 52 | | 100 | 0 |
| **18** | CNCLEP00031147 | Coleophoridae | Coleophora cornivorella | 76 | 65 | 67 | | 100 | 0 |
| **19** | CNCLEP00031148 | Coleophoridae | Coleophora latronella | 76 | 58 | 64 | | 99.2 | 5 |
| **20** | CNCLEP00031099 | Coleophoridae | Coleophora limosipennella | 68 | 66 | 88 | | 100 | 0 |
| **21** | CNCLEP00022601 | Coleophoridae | Coleophora limosipennella | 68 | 67 | 48 | | 100 | 13 |
| **22** | CNCLEP00031150 | Coleophoridae | Coleophora duplicis group | 66 | 73 | 54 | | 100 | 0 |
| **23** | CNCLEP00001026 | Coleophoridae | Coleophora wyethiae | 79 | No Pyrosequence | | | | |
| **24** | CNCLEP00001025 | Coleophoridae | Coleophora wyethiae | 79 | 60 | 73 | | 100 | 0 |
| **25** | CNCLEP00031151 | Coleophoridae | Coleophora duplicis group | 56 | 61 | 82 | | 100 | 0 |
| **26** | CNCLEP00019885 | Coleophoridae | Coleophora duplicis group | 56 | 90 | 50 | | 97.7 | 0 |
| **27** | CNCLEP00019886 | Coleophoridae | Coleophora paludoides | 71 | 56 | 66 | | 100 | 7 |
| **28** | CNCLEP00019888 | Coleophoridae | Coleophora persimplexella | 58 | 80 | 105 | | 100 | 0 |
| **29** | CNCLEP00031152 | Coleophoridae | Coleophora duplicis group | 58 | 81 | 73 | | 100 | 0 |
| **30** | CNCLEP00019889 | Coleophoridae | Coleophora duplicis group | 58 | 57 | 80 | | 99.2 | 0 |
| **31** | CNCLEP00019890 | Coleophoridae | Coleophora quadruplex | 75 | 54 | 60 | | 100 | 13 |
| **32** | CNCLEP00031097 | Coleophoridae | Coleophora rosacella | 75 | 57 | 70 | | 100 | 0 |
| **33** | CNCLEP00031153 | Coleophoridae | Coleophora rosaevorella | 65 | No Pyrosequence | | | | |
| **34** | CNCLEP00019892 | Coleophoridae | Coleophora rosaevorella | 65 | 83 | 4 | | 98.5 | 0 |
| **35** | CNCLEP00019891 | Coleophoridae | Coleophora rupestrella | 57 | 71 | 44 | | 100 | 12 |
| **36** | CNCLEP00031154 | Coleophoridae | Coleophora salicivorella | 66 | 67 | 54 | | 100 | 7 |
| **37** | CNCLEP00031098 | Coleophoridae | Coleophora laricella | 66 | 68 | 75 | | 100 | 0 |
| **38** | CNCLEP00031155 | Coleophoridae | Coleophora salinoidella | 67 | 68 | 65 | | 100 | 0 |
| **39** | CNCLEP00019894 | Coleophoridae | Coleophora salinoidella | 97 | 63 | 64 | | 99.2 | 3 |
| **40** | CNCLEP00019895 | Coleophoridae | Coleophora seminella | 66 | 49 | 71 | | 100 | 8 |
| **41** | CNCLEP00031095 | Coleophoridae | Coleophora serratella | 66 | 59 | 69 | | 100 | 0 |
| **42** | CNCLEP00031157 | Coleophoridae | Coleophora sexdentatella | 54 | 55 | 75 | | 100 | 0 |
| **43** | CNCLEP00019896 | Coleophoridae | Coleophora sexdentatella | 54 | 71 | 58 | | 100 | 0 |
| **44** | CNCLEP00019897 | Coleophoridae | Coleophora simulans | 56 | 57 | 64 | | 100 | 6 |
| **45** | CNCLEP00031158 | Coleophoridae | Coleophora sparsipulvella | 79 | 69 | 58 | | 99.2 | 0 |
| **46** | CNCLEP00019898 | Coleophoridae | Coleophora sparsipulvella | 79 | 60 | 89 | | 100 | 0 |
| **47** | CNCLEP00031096 | Coleophoridae | Coleophora spinella | 79 | 88 | 45 | | 100 | 0 |
| **48** | CNCLEP00031160 | Coleophoridae | Coleophora triplicis | 71 | 72 | 79 | | 100 | 0 |
| **49** | CNCLEP00019900 | Coleophoridae | Coleophora triplicis | 71 | 53 | 62 | | 100 | 12 |
| **50** | CNCLEP00022602 | Coleophoridae | Coleophora vacciniivorella | 56 | 81 | 48 | | 96.9 | 0 |
| **51** | CNCLEP00031159 | Coleophoridae | Coleophora versurella | 71 | 77 | 69 | | 99.2 | 0 |
| **52** | CNCLEP00019899 | Coleophoridae | Coleophora versurella | 71 | 44 | 83 | | 100 | 0 |
| **53** | CNCLEP00031146 | Coleophoridae | Coleophora juglandella | 66 | 60 | 83 | | 100 | 0 |
| **54** | CNCLEP00001125 | Coleophoridae | Coleophora rosaevorella | 79 | 58 | 91 | | 100 | 0 |
| **55** | CNCLEP00031053 | Coleophoridae | Coleophora rosaevorella | 79 | 61 | 58 | | 100 | 8 |
